# A review of new developments in the Friedel–Crafts alkylation – From green chemistry to asymmetric catalysis

**DOI:** 10.3762/bjoc.6.6

**Published:** 2010-01-20

**Authors:** Magnus Rueping, Boris J Nachtsheim

**Affiliations:** 1 Institute of Organic Chemistry, RWTH Aachen, Landoltweg 1, 52074 Aachen, Germany; 2 Department of Biological Chemistry and Molecular Pharmacology, Harvard Medical School, 240 Longwood Avenue, Boston, MA 02115, USA

**Keywords:** allyl alcohols, arene, asymmetric Friedel–Crafts reaction, benzyl alcohols, Friedel–Crafts alkylation, green chemistry, hydroalkylation, hydroarylation, Lewis-acid catalysis, propargyl alcohols

## Abstract

The development of efficient Friedel–Crafts alkylations of arenes and heteroarenes using only catalytic amounts of a Lewis acid has gained much attention over the last decade. The new catalytic approaches described in this review are favoured over classical Friedel–Crafts conditions as benzyl-, propargyl- and allyl alcohols, or styrenes, can be used instead of toxic benzyl halides. Additionally, only low catalyst loadings are needed to provide a wide range of products. Following a short introduction about the origin and classical definition of the Friedel–Crafts reaction, the review will describe the different environmentally benign substrates which can be applied today as an approach towards greener processes. Additionally, the first diastereoselective and enantioselective Friedel–Crafts-type alkylations will be highlighted.

## Introduction

**Scheme 1:**
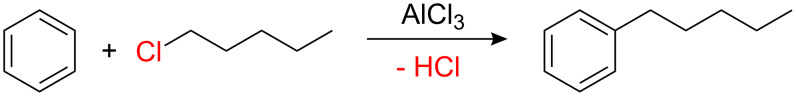
AlCl_3_-mediated reaction between amyl chloride and benzene as developed by Friedel and Crafts.

In 1887 Charles Friedel and James Mason Crafts isolated amylbenzene after the treatment of amyl chloride with AlCl_3_ in benzene (Scheme 1) [1]. This was not only one of the first descriptions of a Lewis acid used in organic synthesis but also the first example of what was later to be called Friedel–Crafts alkylation (FC alkylation) after its inventors. Today Friedel–Crafts alkylations remain the method of choice for the alkylation of arenes and heteroarenes.

**Figure 1:**
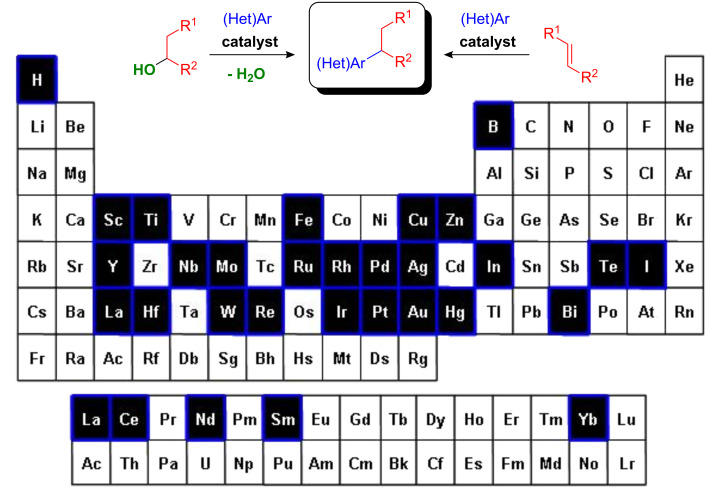
Most often used metal salts for catalytic FC alkylations and hydroarylations of arenes.

Over the intervening years many other Lewis acids including BF_3_, BeCl_2_, TiCl_4_, SbCl_5_ or SnCl_4_ have been described as catalysts for the FC alkylation. Furthermore, strong Brønsted-acids including sulfuric acid, hydrofluoric acid or super acids such as HF•SbF_5_ and HSO_3_F•SbF_5_ have also been shown to accelerate this transformation. Despite the great importance of the Friedel–Crafts alkylation for organic synthesis it has major drawbacks since stoichiometric or super stoichiometric amounts of a Lewis acid or Brønsted acid and toxic alkyl halides have to be utilized leading to vast amounts of salt side products. With the need for more environmentally and economically benign processes, the development of FC reactions using only catalytic amounts of a metal or acid catalyst would be highly desirable. In addition, the substitution of the alkyl chlorides by other, less toxic, alkylating reagents such as alcohols would be a major improvement as water would be the only side product. Beyond this, the use of activated double bonds and styrenes would be even more efficient as no side products are to be expected. However, good ideas always need time to develop and grow and thus it is not surprising that it took more than 100 years from the initial invention of Friedel and Crafts in 1887 until the first catalytic FC alkylations with alcohols and styrenes as alkylating reagents were developed. Initial attempts in 1996 using Sc(OTf)_3_ and soon after with Mo(CO)_6_ as Lewis acid catalysts were consequently followed by a multitude of new methods employing a variety of Lewis- and Brønsted acids with decreasing catalyst loadings and in consequence increasing efficiencies (Figure 1).

With regard to the electrophiles employed in the FC alkylation protected or activated alcohols and styrenes have been found to be suitable alkylating reagents giving access to many functionalized arenes including 1,1-diarylalkanes, allyl- and prop-2-ynylbenzenes. More recently diastereoselective and enantioselective Friedel–Crafts alkylations have been developed. In this review we intend to give an overview of the important developments that have primarily emerged over the last decade.

## Review

### The FC alkylation with benzyl alcohols – An efficient approach to 1,1-diarylalkanes

**Figure 2:**
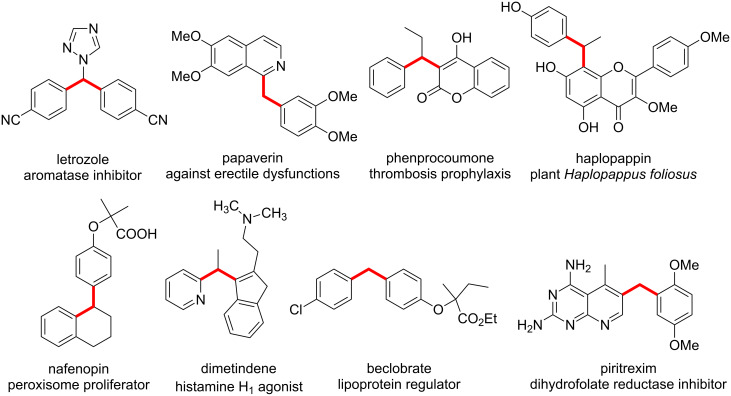
1,1-diarylalkanes with biological activity.

1,1-Diarylalkanes are important building blocks for the synthesis of many pharmaceuticals, agro- and fine chemicals (Figure 2).

**Scheme 2:**
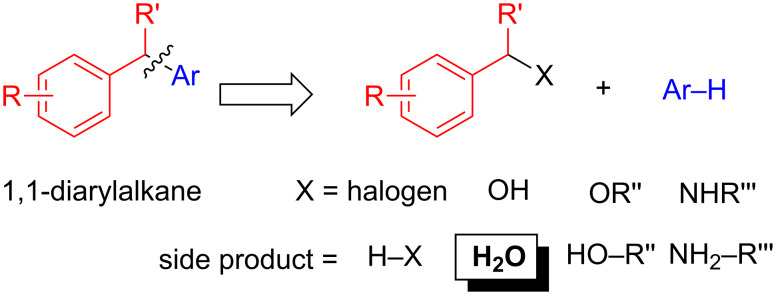
Alkylating reagents and side products produced.

Traditionally, 1,1-diarylalkanes have been prepared from benzyl halides under Friedel–Crafts conditions using stoichiometric amounts of a Lewis acid, such as AlCl_3_. With the need for more environmentally and economically benign processes, the Friedel–Crafts-type synthesis of 1,1-diarylalkanes using catalytic amounts of a metal or acid catalyst and more environmental friendly benzylation reagents are highly desirable. To this end substantial progress has been made and different benzyl halide substitutes, including free and protected alcohols as well as tosylamides have been introduced (Scheme 2).

In particular benzyl alcohols have become a valuable alternative. Due to their availability, lower toxicity, and the fact that only stoichiometric amounts of water are generated as the side product the FC alkylation with benzyl-, allyl- and propargyl alcohols presented a first and important step toward an environmental friendly process.

**Scheme 3:**
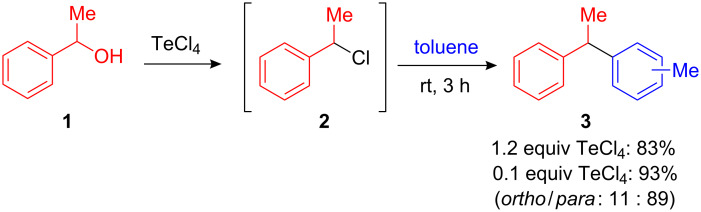
Initially reported TeCl_4_-mediated FC alkylation of 1-penylethanol with toluene.

In 1986, Uemura et al. investigated the chlorination of benzyl- and alkyl alcohols mediated by SeCl_4_ and TeCl_4_. While the reaction performed in non-aromatic solvents yielded the desired benzyl chlorides in good yields, an unexpected side reaction was observed in aromatic solvents such as toluene resulting in the 1,1-diarylalkane **3** in 83% yield (Scheme 2). The authors explained this observation with a chlorination of 1-phenylethanol **1** and subsequent FC alkylation of the formed benzyl chloride and toluene. However, more surprisingly the reaction yield could be improved to 93% if only catalytic amounts (10 mol%) of TeCl_4_ were present (Scheme 3) [2].

Although the reaction was found by accident, this was probably the first description of a catalytic FC alkylation utilizing a benzyl alcohol.

**Scheme 4:**
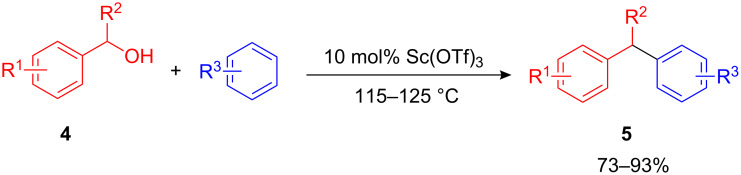
Sc(OTf)_3_-catalyzed FC benzylation of arenes.

The first systematic investigations of catalytic FC benzylations were performed independently in 1996 and 1997 by Fukuzawa [3–4] and Shimizu et al. [5]. While the latter used 10 mol% Mo(CO)_6_ as the Lewis acid catalyst under the strict exclusion of air and moisture, the Fukuzawa group utilized Sc(OTf)_3_ as a water and air tolerant catalyst. Various arenes, including benzene, *p*-xylene, or mesitylenes were alkylated with benzyl alcohols **4** to afford the desired 1,1-diarylalkanes **5** in high yields (Scheme 4).

Other rare-earth trifluormethanesulfonates such as Nd(OTf)_3_ Yb(OTf)_3_ and Sm(OTf)_3_ showed similar reactivities and the reaction was later also performed in the ionic liquids [BMIM][OTf] and [BMIM][PF_6_] [6]. Next to benzyl alcohol, allyl alcohols, dibenzylethers as well as arenecarbaldehydes and their corresponding acetals have been used as electrophilic component [7].

**Scheme 5:**
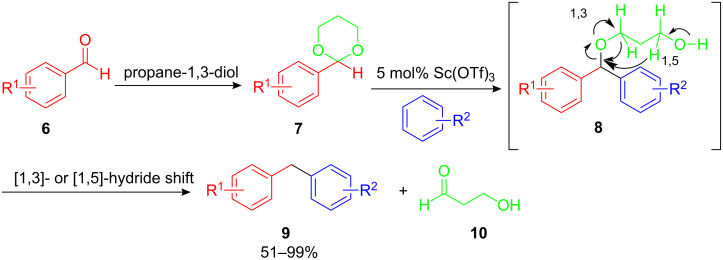
Reductive FC alkylation of arenes with arenecarbaldehydes.

While FC alkylations with allyl alcohols and benzyl ethers are likely to have the same carbocationic reaction intermediate, the FC alkylation with arenecarbaldehydes **6** has to be different (Scheme 5). Mechanistic investigations revealed that propanediol is necessary for this reaction to proceed. In the first step of the reaction sequence a Lewis acid catalyzed acetalization of the aldehyde occurs and the acetal **7** is formed. The following nucleophilic attack of the arene yields diphenyl-substituted ether **8** as an intermediate which subsequently undergoes an intramolecular [1,3]- or [1,5]-hydride shift resulting in the desired diarylmethanes **9** in good yields. Electron donating and electron withdrawing functional groups of aldehyde moiety are tolerated in this reductive Friedel–Crafts alkylation procedure. However, there is no clear correlation between the electron deficiency of the arenecarbaldehyde and the reaction yield [4].

Further mechanistic investigations were performed with 1,3-propanediol-*1*,*1*,*3*,*3*-*d*_4_ in order to provide more information about the observed hydride shift. Indeed deuterium was incorporated into the benzylic position and monodeuterated diphenylmethane was obtained in over 95% yield. This result and the fact that 3-hydroxypropanal **10** is a major side product strongly supports the mechanism of this reductive FC alkylation reaction.

Soon after the fundamental work by Fukuzawa and Shimizu, many catalytic FC benzylations using benzyl alcohols have been developed. These utilize for instance Cl_2_Si(OTf)_2_, Hf(OTf)_4_ [8], Yb(OTf)_3_, La(OTf)_3_ [9], InCl_3_ [10–11], NbCl_5_ [12], heterobimetallic Ir-Sn-catalysts [13–14], H-mont [15], [CpMoCl(CO)_3_]/*o*-chloranil [16], strong Brønsted acids [17–19] calix[6]arene sulfonates [20] or molecular iodine [21–22] as catalysts.

**Scheme 6:**
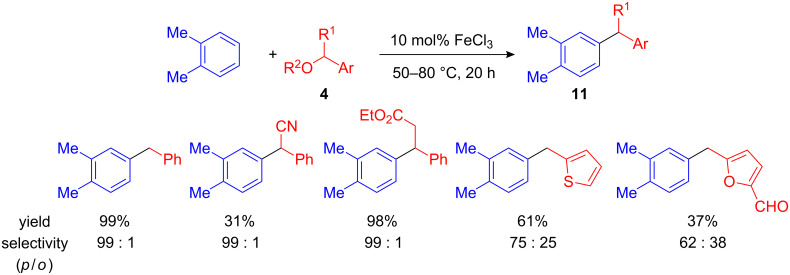
Iron(III)-catalyzed FC benzylation of arenes and heteroarenes.

In 2005 Beller et al. systematically tested the activity of various Lewis- and Brønsted acids in FC benzylations. They found that late transition metals such as HAuCl_4_ [23], IrCl_3_, [MesW(CO)_3_], RhCl_3_, H_2_PdCl_4_, H_2_PtCl_6_ [24] and FeCl_3_ [25] were the most effective. FeCl_3_, in particular, is an attractive alternative to rare-earth triflates since it is non-toxic, cheap and readily available. Different benzyl alcohols and acetates (Scheme 6, R^1^ = H) and 1-aryl alcohols (R^1^ = Me) are tolerated in the reaction if 10 mol% FeCl_3_ catalyst are applied. Even fairly unstable thiophene- and furan-2-carbaldehyde derived benzyl alcohols, cyano(phenyl)methyl acetate or 3-hydroxy-3-phenylpropanoates and benzyl methyl ethers have been successfully applied as benzylation reagents [26].

**Scheme 7:**
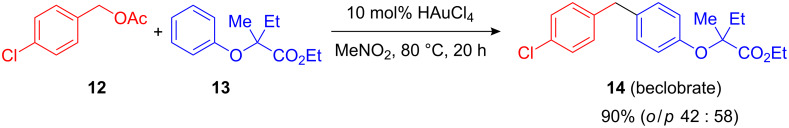
A gold(III)-catalyzed route to beclobrate.

Later, the same authors used gold(III) as a catalyst for an efficient one-pot synthesis of beclobrate, a well known fibric acid derivative with a potent hypolipidemic activity [23]. The straightforward synthesis was accomplished by reaction of readily available *p*-chlorobenzyl acetate (**12**) with 2-methyl-2-phenoxybutyrate **13** to give beclobrate (**14**) in 90% yield. However, 10 mol% of HAuCl_4_ had to be used (Scheme 7).

**Scheme 8:**
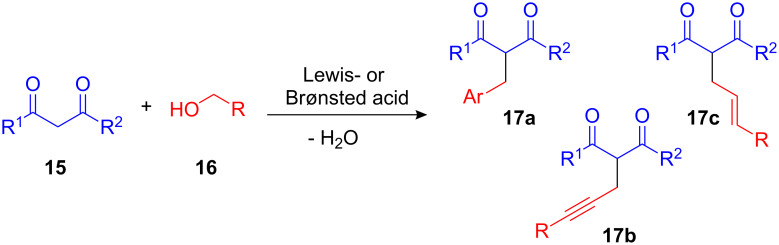
Catalytic FC-type alkylations of 1,3-dicarbonyl compounds.

The Lewis- and Brønsted acid catalyzed activation of benzyl alcohols and derivatives is not only restricted to the Friedel–Crafts alkylation and the application of arenes but can additionally be extended to other nucleophiles. In particular, mono-substituted 1,3-diketones **17** and their derivatives are of great interest. Given that the alkylation typically requires stoichiometric amounts of a base and a toxic alkyl halide the development of an efficient environmentally benign route to 2-alkylated pentanediones **17a–c** employing simply benzyl-, allyl- or propargyl alcohols **16** as alkylating reagents represented a valuable advancement (Scheme 8) [27–45].

**Scheme 9:**
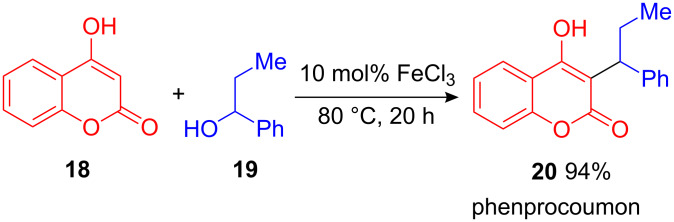
Iron(III)-catalyzed synthesis of phenprocoumon.

An example of this procedure represents the effective iron(III)-catalyzed one-step synthesis of phenprocoumon (**20**), an anticoagulant of the warfarin-class that is widely used in thrombosis prophylaxis. Simply starting from 4-hydroxycoumarin (**18**) and 1-phenylpropan-1-ol (**19**), phenprocoumon (**20**) was obtained in 94% yield (Scheme 9) [27].

**Scheme 10:**
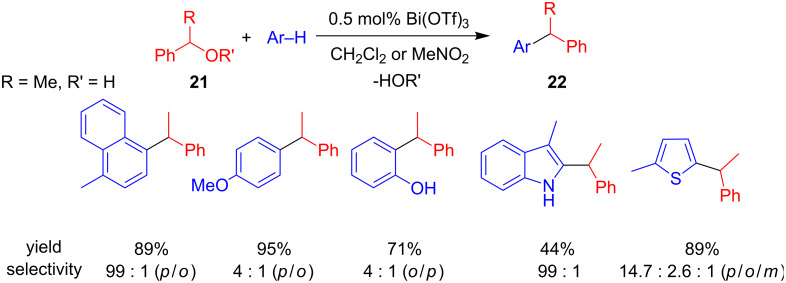
Bi(OTf)_3_-catalyzed FC alkylation of benzyl alcohols developed by Rueping et al.

As a suitable alternative to transition metals, bismuth salts have emerged as cheap, non-toxic, and readily available catalysts with Lewis acidic properties. Given that certain bismuth salts are compatible with air and moisture, the Rueping group decided to examine the bismuth-catalysed arylation and alkylation of benzyl alcohols. With small amounts of Bi(OTf)_3_ (0.5 mol%) they were able to benzylate arenes bearing methyl, methoxy or hydroxy functionalities as well as heteroarenes, including thiophenes or 3-methylindole with a good *para*/*ortho*/*meta* regioselectivity (Scheme 10).

**Scheme 11:**
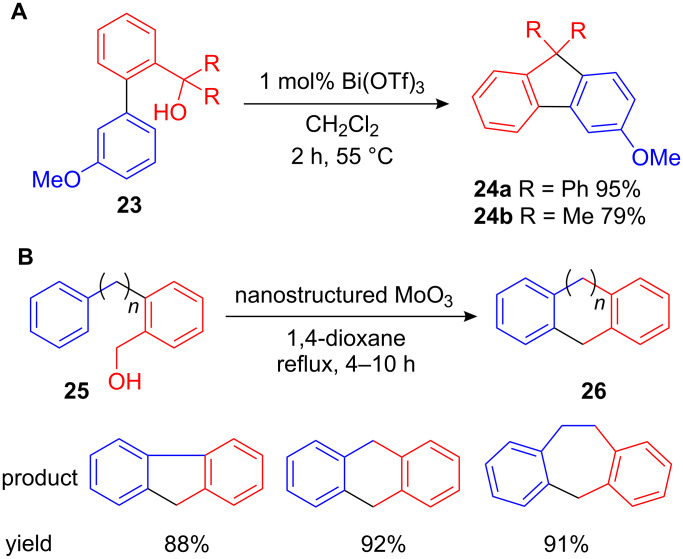
**(A)** Bi(OTf)_3_-catalyzed intramolecular FC alkylation as an efficient route to substituted fulvenes. **(B)** Nanostructured MoO_3_ mediated intramolecular FC alkylation.

In addition, they successfully used benzyl acetates, 1-phenylethanol and β-hydroxy-substituted benzyl alcohols as alkylating reagents. Furthermore, an efficient intramolecular variant of this procedure starting from biaryl benzyl alcohol **23** led to substituted fluorenes **24** which have shown to be valuable scaffolds for blue light emitting polymers (Scheme 11A) [46]. A similar route to fluorenes and other annulated cycloalkanes **26** was subsequently developed utilizing nanostructured MoO_3_ (Scheme 11B) [47–48].

**Scheme 12:**
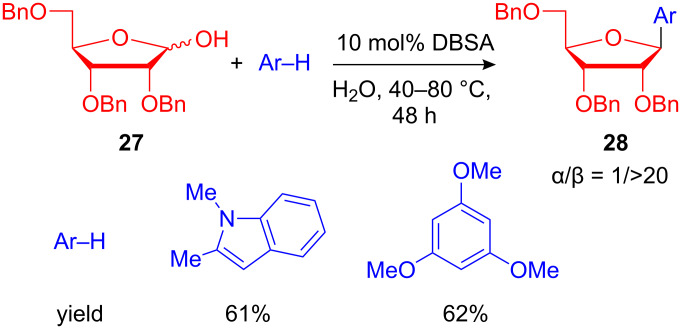
FC-type glycosylation of 1,2-dimethylindole and trimethoxybenzene.

Recently, Kobayashi et al. reported a dehydrative nucleophilic substitution of benzyl alcohols in water employing a dodecylbenzenesulfonic acid (DBSA) as a surfactant-type Brønsted acid catalyst. With this green methodology a variety of carbon- and heteroatom-centered nucleophiles were effectively applied resulting numerous diarylmethanes and 3-substituted indoles. Moreover, this method could be extended to the C-glycosylation of 1-hydroxysugars and the products **28** were obtained in high yields and with remarkable anomeric ratios (Scheme 12) [49].

**Scheme 13:**
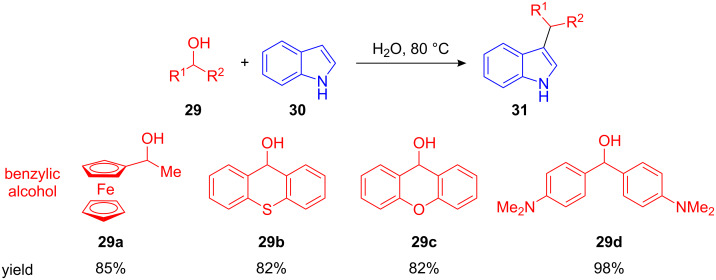
FC alkylation with highly reactive ferrocenyl- and benzyl alcohols. The reaction proceeds even without Lewis acids just “on water”.

Recently, Cozzi et al. showed that FC benzylations can proceed even without adding a Lewis acid catalyst, just “on water” at 80 °C. However, this method is restricted to reactive heteroarenes such as indole (**30**), pyrrole and nucleophiles including azides or acetylacetonates. Moreover, only highly reactive ferrocenyl alcohols **29a** or benzhydrols **29b–d**, which result in highly stabilized carbocations upon elimination, can be used in this procedure (Scheme 13) [50].

**Scheme 14:**
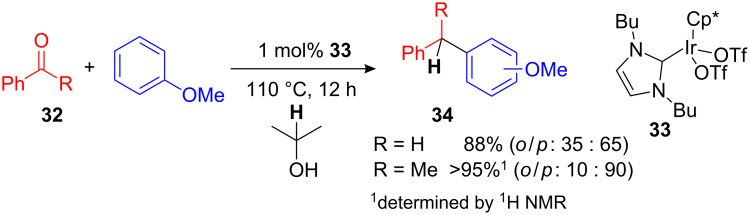
Reductive FC alkylation of arenes with benzaldehyde and acetophenone catalyzed by the Ir-carbene complex **33**.

An interesting domino reduction–alkylation procedure was recently developed by Peris et al. employing a versatile Ir-Cp*-catalyst **33** (Scheme 14). This *N*-heterocyclic carbene Ir-complex is capable of catalyzing FC alkylations not only with benzyl alcohols and styrenes but also utilizing aldehydes and acetophenones **32**, which are reduced prior to the FC-type alkylation in the same reaction vessel (Scheme 14). Here isopropanol can be used as a simple reducing reagent to afford the desired 1,1-diarylalkanes **34** in high yields after 12 h with just 1 mol% of the Ir-complex **33**. Thus this procedure widens the scope of electrophiles that can be used in environmental benign FC processes [51].

### Hydroarylations for the synthesis of 1,1-diarylalkanes

**Scheme 15:**
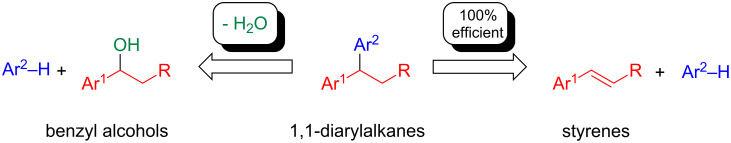
Formal synthesis of 1,1-diarylalkanes from benzyl alcohols and styrenes.

The Friedel–Crafts benzylation of arenes using benzyl alcohols was discussed in the previous chapter. Even though it renders a convenient and environmental benign approach to 1,1-diarylalkanes, there is still one stoichiometric side product formed during this transformation, namely water. Waste water treatment is an ongoing and expensive issue in large scale chemical processes; therefore, methodologies without the formation of water or other by-products would be even more desirable. As such, hydroarylations and hydroalkylations with a theoretical atom efficiency of 100% would solve these issues. The Friedel–Crafts-type 1,4-addition of arenes to unsaturated carbonyl compounds and their derivatives can be considered as Michael reactions. They have been extensively reviewed elsewhere and will be not discussed in this chapter [52–55]. For the synthesis of 1,1-diarylalkanes, efficient hydroarylation procedures employing styrenes and other activated double bonds are needed (Scheme 15). Due to their availability, styrenes are most suitable substrates for potential hydroarylation methods and a fast synthesis of compound libraries should be feasible.

**Scheme 16:**
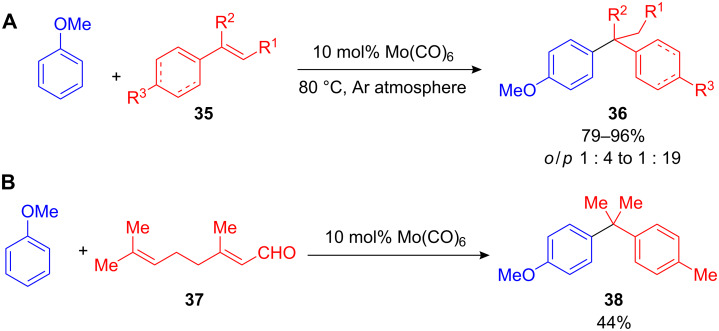
**(A)** Mo-catalyzed hydroarylation of styrenes and cyclohexenes. **(B)** Hydroalkylation–cyclization cascade leading to 1,1-diarylalkane **38** from the linear precursor citral (**37**).

Although, the Murai reaction, the Ru-catalyzed *ortho*-alkylation of acetophenones with alkenes has been known since 1993 [56–57], the direct substitution of arenes with styrenes is a development that has emerged in the last decade. Within this context, Shimizu and co-workers discovered a Lewis-acid-catalyzed substitution of arenes with olefins, such as styrene, α- and β-methylstyrene or cyclohexenes **35** (Scheme 16A) [5]. Various transition-metal compounds such as Mo(CO)_6_, MoCl_5_, W(CO)_6_, H_2_IrCl_5_, Sc(OTf)_3_ as well as several Brønsted acids were described, with Mo(CO)_6_ being the most efficient. Like other FC alkylations, the *para*-substituted product **36** was highly preferred. Interestingly, the hydroalkylation of anisole with citral (**37**) did not result in the expected alkyl-substituted anisole derivative. Instead, the diarylalkane **38** was obtained in 44% yield, most likely through a FC alkylation, cationic cyclization reaction cascade (Scheme 16B).

**Scheme 17:**
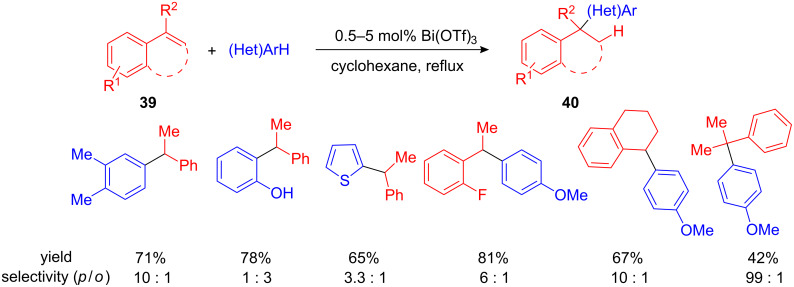
Bi(III)-catalyzed hydroarylation of styrenes with arenes and heteroarenes.

Unfortunately 10 mol% of expensive, toxic and air- and moisture sensitive Mo(CO)_6_ was necessary for a successful transformation. It took almost nine years from this first discovery until Beller et al. and Rueping et al. developed Fe(III)- and Bi(III)-catalyzed hydroarylations of arenes [58–59]. Although, FeCl_3_ is cheap, non-toxic and readily available, a high catalyst loading (10 mol%) was necessary to obtain complete conversion. In comparison, 0.5 mol% of Bi(OTf)_3_ were sufficient to give the desired 1,1-diarylalkanes after short reaction times in good to excellent yield and with good *ortho*/*para* selectivity. Although different arenes and heteroarenes, including thiophene can be efficiently alkylated, furans did not result in the desired products. In addition to styrene, dihydronaphthalene and α-methylstyrene could be used as electrophiles. In the latter case, the formation of a quaternary carbon atom was possible. Although the product was isolated in lower yields (Scheme 17). Subsequently this method was expanded to other nucleophiles, such as 1,3-diketones [60].

**Scheme 18:**
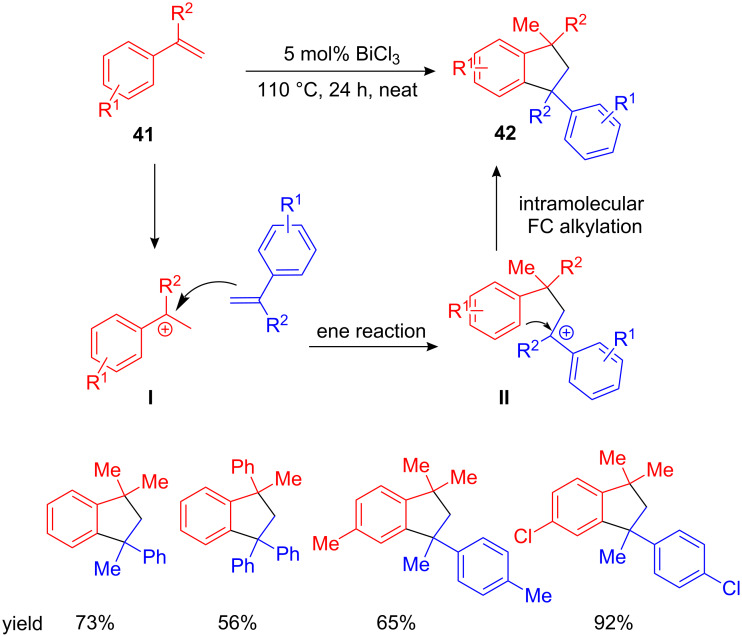
BiCl_3_-catalyzed ene/FC alkylation reaction cascade – A fast access to highly arylated dihydroindenes.

Mirroring the Bi(OTf)_3_-catalyzed method, Hua et al. developed a BiCl_3_-catalyzed synthesis of 1,1-diarylalkanes starting from electron-rich arenes and styrenes. Additionally, they found that heating of styrene **41** in the presence of catalytic amounts of BiCl_3_ yielded substituted dihydroindenes **42** as a result of styrene dimerization. This reaction may proceed via an intermolecular ene reaction between styrene and the carbocationic intermediate **I** followed by an intramolecular FC alkylation of the resulting carbocationic 1,3-diarylpropane **II** (Scheme 18) [61].

Next to the described procedures many related intermolecular FC-type alkylations with styrenes and activated double bonds have been developed using, for instance, InCl_3_/SiO_2_ [11], Iodine [62], Ir(III) [51], AuCl_3_/AgSbF_6_ [63], AuCl [64], and PtCl_2_ [65].

**Scheme 19:**
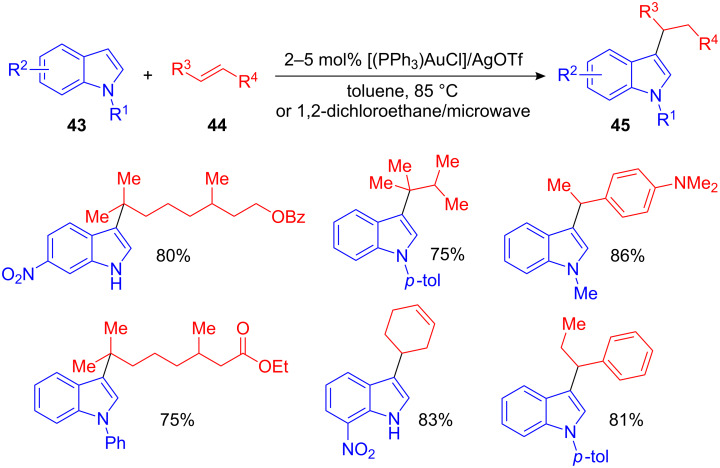
Au(I)/Ag(I)-catalyzed hydroarylation of indoles with styrenes, aliphatic and cyclic alkenes.

Within the row of heteroarenes, indole is one of the most important structural motifs due to its abundance in biologically active small molecules; thus their hydroarylations are particularly useful. A gold(I)-catalyzed hydroarylation of indoles with styrenes as well as with aliphatic and cyclic alkenes was developed by Che et al. [64]. [AuCl(PPh_3_)]/AgOTf was the catalyst system of choice and the reaction was, depending on the substrate, performed under thermal or microwave-assisted conditions. The wide range of alkene substrates and the low catalyst amounts render this method a highly efficient and convenient synthesis of 3-functionalized indole derivatives (Scheme 19).

An elegant PtCl_2_-catalyzed intramolecular alkylation of indoles with inactivated alkenes has been developed by Widenhoefer et al. [65]. In addition to the transition metal Pt(II), catalytic amounts of hydrochloric acid were necessary to obtain the desired 2,3-annulated indoles in sufficient yields. By employing optically pure axial chiral phosphane ligands, an enantioselective version of this transformation was developed which provided the products with high enantioselectivities.

**Scheme 20:**
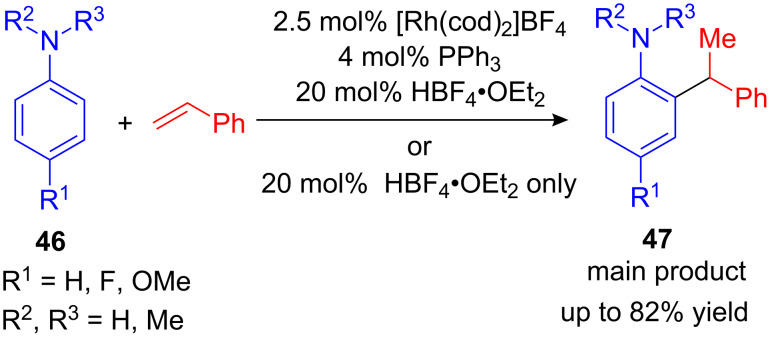
First transition-metal-catalyzed *ortho*-hydroarylation developed by Beller et al.

Beside indoles, anilines have gained much attention as target for hydroarylation reactions. However, the main issues for FC alkylations of anilines are the deactivation of the catalyst due to coordination of the primary amine and/or concurrent hydroamination reactions [66]. Nevertheless, Beller et al. developed a valuable method to overcome these limitations. In 1999 they described the transition metal catalyzed hydroarylation of anilines **46** with styrenes using a cationic Rh-complex [67]. Depending on the aniline derivative, a combination of 2.5 mol% [Rh(cod)_2_]BF_4_, 4 mol% PPh_3_ and HBF_4_•OEt_2_ was necessary for sufficient reactivity; yet electron-rich and *N*-alkylated anilines react without a metal catalysis in the presence of catalytic amounts of HBF_4_ (Scheme 20). About the same time a Ru_3_(CO)_12_-catalyzed hydroarylation of anilines was reported as well [68].

**Scheme 21:**
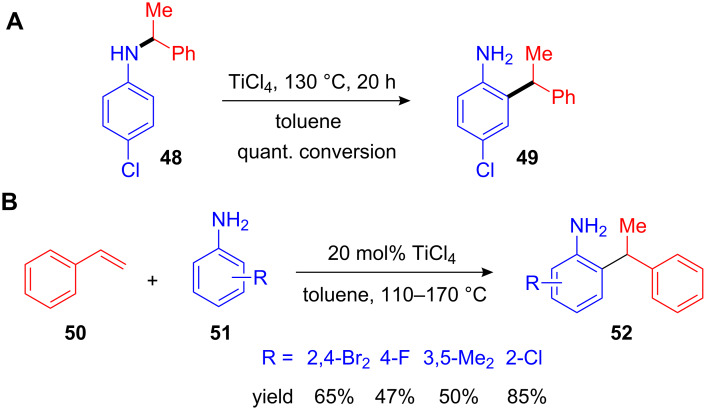
**(A)** Ti(IV)-mediated rearrangement of an *N*-benzylated aniline to the corresponding *ortho*-alkylated aniline. **(B)** *ortho*-Arylation of anilines with styrenes in the presence of TiCl_4_.

Ackermann et al. attempted to develop a Ti(IV)-catalyzed hydroamination of anilines with olefins, and surprisingly discovered that a mixture of hydroamination and *ortho*-hydroarylation products was formed. Upon further heating of the secondary amine **48** in the presence of TiCl_4_ the *ortho*-arylated aniline **49** was formed quantitatively (Scheme 21A). Reaction conditions were improved and with 20 mol% of TiCl_4_ a variety of electron-rich and electron-poor anilines **51** and styrenes **50** were utilized for giving diverse *ortho*-benzylated anilines **52** in moderate to good yields (Scheme 21B) [69].

Almost simultaneously, an acid-catalyzed *ortho*-arylation of aniline employing the strong Brønsted acid PhNH_3_B(C_6_F_5_)_4_•Et_2_O was developed by Bergman et al. indicating the close relationship between strong Brønsted acid- and transition-metal catalysis [70].

**Scheme 22:**
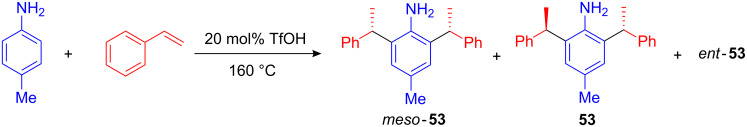
Dibenzylation of aniline gives potentially useful amine-based ligands in a one-step procedure.

A double *ortho*-hydroarylation of anilines with styrenes was presented by Coates and co-workers giving a mixture of **53** and *ent*-**53** and the corresponding *meso* compound. In combination with enantioselective separation techniques, such as HPLC, or upon resolution with chiral Brønsted acids this method provides valuable *ortho*-chiral anilines that could be used as valuable optical active ligands for enantioselective transition-metal catalysis (Scheme 22) [71].

To the best of our knowledge, efficient *ortho*-benzylations of anilines with benzyl alcohols as alkylating reagents have yet to be described [15].

### Catalytic Friedel–Crafts allylations

**Scheme 23:**
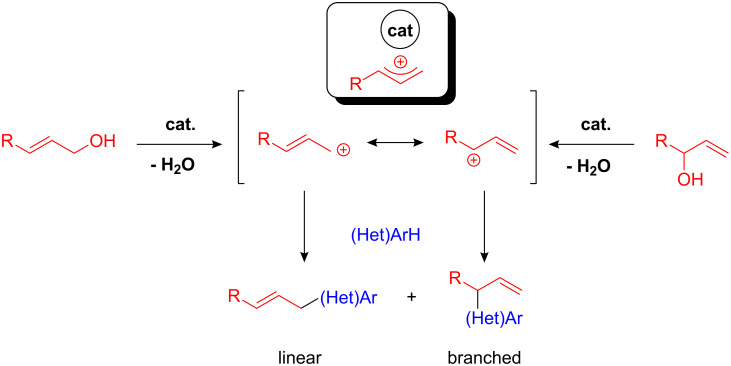
FC-type alkylations with allyl alcohols as alkylating reagents – linear vs. branched product formation.

Allylated arenes represent attractive precursors for organic synthesis as there are several possibilities for further transforming the exocyclic double bonds. Typically, in transition-metal-catalyzed allylation reactions reactive, metal-coordinated allyl cations are formed which may lead to linear and branched products, whereby the product ratio is dependent on the catalyst employed (Scheme 23). To date, only few FC-type allylations with environmentally benign allylating reagents, such as allyl alcohols have been reported.

**Scheme 24:**
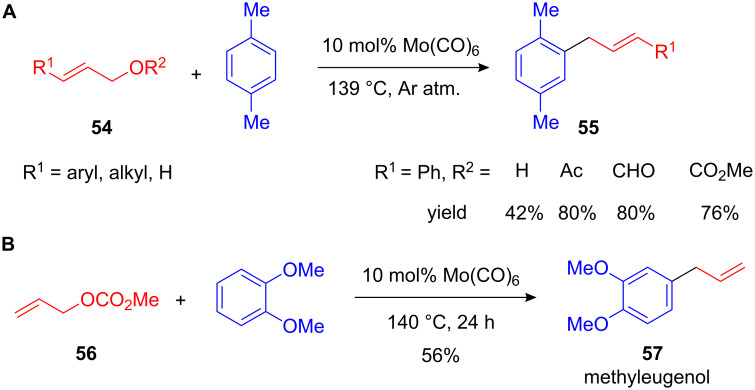
**(A)** First catalytic FC allylation and cinnamylation using allyl alcohols and its derivatives. **(B)** Efficient Mo-catalyzed synthesis of methyleugenol.

Here Shimizu and co-workers did seminal work by developing a Mo(CO)_6_- and W(CO)_6_-catalyzed allylation and cinnamylation of electron-rich arenes. Employing, for instance, *p*-xylene or toluene and cinnamyl alcohols or -carbonates **54** in the presence of 10 mol% of Mo(CO)_6_ the desired allylated arenes **55** could be isolated in good yields (Scheme 24A) [72]. As application of this methodology, methyleugenol **57**, an ingredient in many spices and essential oils, could be synthesized in one step starting from allyl carbonate **56** and 1,2-dimethoxybenzene (Scheme 24B). A similar method using a diruthenium complex was developed by Hidai and co-workers [73]. The reaction conditions of both methods were still harsh and the use of Mo(CO)_6_ as a catalyst required exclusion of air and moisture. Moreover, the reactive allyl carbonates or acetates had to be applied due to the easier activation as better leaving groups. Hence, improved procedures with less sensitive and cheaper catalysts as well as unprotected alcohols had to be developed.

**Scheme 25:**
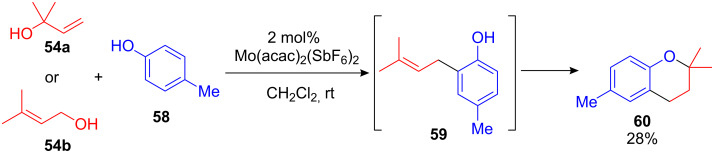
FC allylation/cyclization reaction yielding substituted chromanes.

One of the first examples of a catalytic FC alkylation of arenes with unprotected allyl- and cinnamyl alcohols was developed by Kočovský et al. in 1999 [74–75]. Employing small amounts of a Mo(IV)-complex, allyl alcohols could be substituted with electron-rich arenes such as phenol and anisol. Interestingly, the application of the in situ generated Mo(IV)-catalyst Mo(acac)_2_(SbF_6_)_2_ resulted exclusively in *C*-allylated arenes, while the catalyst precursor Mo(acac)_2_Cl_2_ gave the *O*-allylated phenols as major products. Remarkably the reaction between *p*-cresol **58** and linear or branched allyl alcohols **54a** or **54b** did not give the corresponding alkylated cresol **59**. Instead the chromane **60** was observed in 28% yield (Scheme 25). This reaction has recently been improved and extended by applying MoCl(CO)_3_Cp and [Mo(CO)_3_Cp]_2_ as transition-metal catalysts [76].

**Scheme 26:**
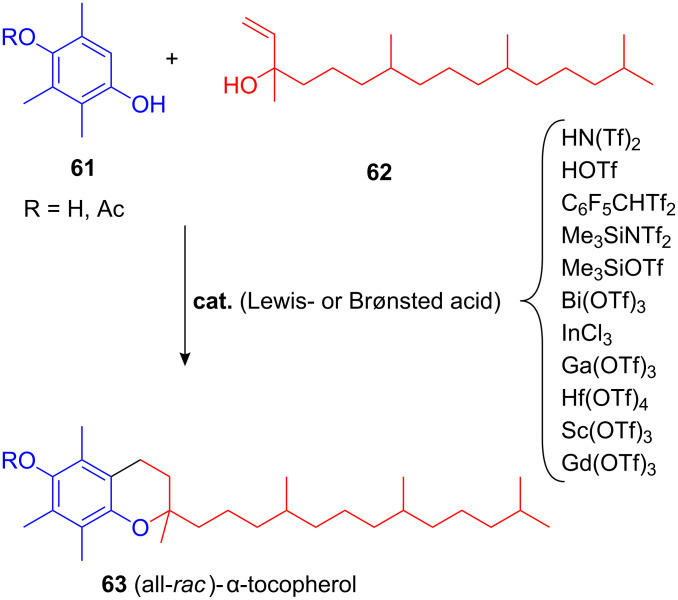
Synthesis of (all*-rac*)-α-tocopherol utilizing Lewis- and strong Brønsted-acids.

A very similar FC allylation/hydroaralyation reaction was subsequently used by other groups for the synthesis of (all-*rac*)-α-tocopherol **63** (Vitamin E) and its more stable acetate derivative starting from the two precursors trimethylhydroquinone **61** and isophytol **62** [77–79]. Strong Brønsted acids as well as various rare-earth metal triflates and silicon-based Lewis acids were used as catalysts (Scheme 26).

**Scheme 27:**
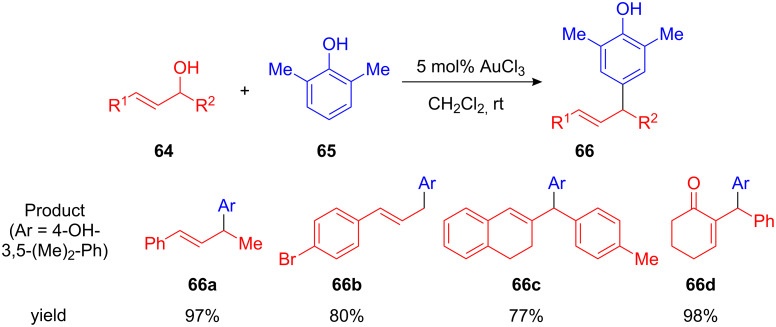
Au(III)-catalyzed cinnamylation of arenes.

Chan and co-workers developed an efficient Au(III)-catalyzed FC arylation of cinnamyl alcohols **64** and electron-rich arenes such as 2,6-dimethylphenol **65**. The authors found that this transformation can be catalyzed by various transition metals and Brønsted acids, including Au(III), Ag(I), In(III), Zn(II), Cu(II) salts or sulfonic acids. AuCl_3_ was the most reactive and was subsequently used for further studies. With 5 mol% of catalyst and performing the reaction at room temperature, the desired allylated arenes and heteroarenes **66** were isolated in good yields after short reaction times. Beside cinnamyl alcohols **66a** and **66b**, 1-arylated allyl alcohols could be used in this transformation giving, for example, the benzylated dihydronaphthalene **66c** and cyclohexanone **66d** in good yield (Scheme 27) [80].

**Scheme 28:**
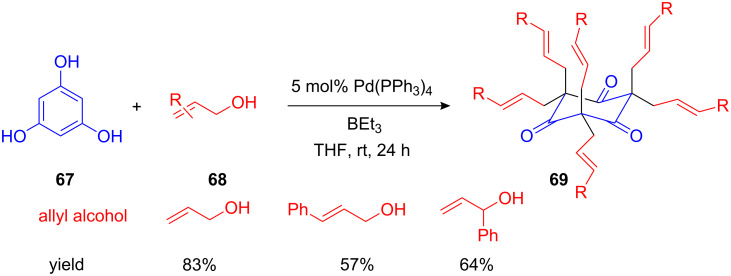
“Exhaustive” allylation of benzene-1,3,5-triol.

As a curiosity, Tamaru et al. reported an “exhaustive” Pd(0)-catalyzed allylation of benzene-1,3,5-triol (**67**), resulting in the highly allylated cyclohexane-1,3,5-trione **69** in high yields. This structure is expected to be a useful precursor for supramolecular architectures with *C*_3_ symmetry (Scheme 28) [81].

It is worth mentioning that in this example as well as in the following Pd-catalyzed allylations an intermediary electrophilic Pd-allyl complex as the reactive allylating species is more likely than the formation of a free allyl cation.

**Scheme 29:**
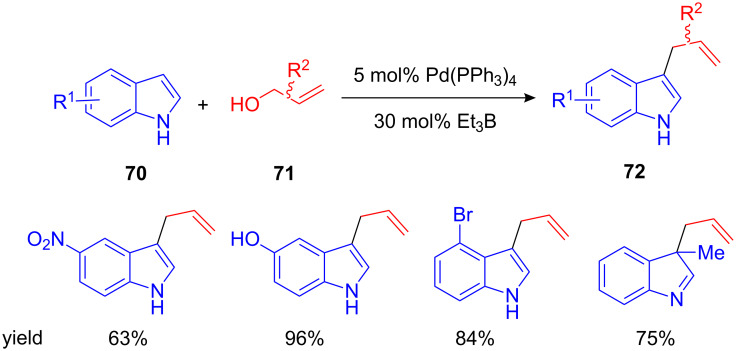
Palladium-catalyzed allylation of indole.

The indole core structure is widely distributed in natural occurring and biologically active molecules and hence, its allylation is of great interest for organic synthesis. It is surprising that it took over eight years from the first description of catalytic allylations of arenes by Shimizu until the first catalytic allylation of indole was described. Here, a combination of Et_3_B and Pd(0) led to an efficient C3-selective allylation [82]. Employing free allyl alcohols and a combination of 5 mol% of Pd(PPh_3_)_4_ and 30 mol% triethylborane as catalyst, the desired 3-allylated indoles were isolated in excellent yields (Scheme 29). Electron withdrawing as well as electron donating groups were well tolerated. Other allylating reagents, such as β-methyl-, α,α-, and γ,γ-dimethylallyl alcohols or cinnamyl alcohol could also be used in this reaction.

**Scheme 30:**
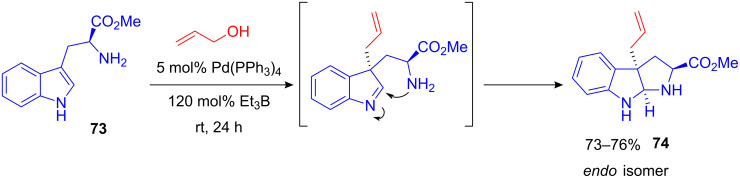
Pd-catalyzed synthesis of pyrroloindoles from L-tryptophane.

Additionally, high selectivity towards the linear allylated indoles was observed. Surprisingly, *N*-allylation did not occur. With L-tryptophan methyl ester (**73**) as the indole moiety, this method led to a convenient stereoselective synthesis of a highly substituted pyrroloindole framework **74** (Scheme 30); however, equimolar amounts of triethylborane were necessary.

An improved FC allylation of indoles with allyl alcohols was developed recently by Breit et al. using self-assembling palladium-phosphane catalysts [83]. Further procedures for the 3-benzylation and allylation of indole employing FeCl_3_ and InBr_3_ have been developed more recently by Yadav and Jana [84–85]. Both procedures are very similar and employ 10 mol% of catalyst. However, the reaction with InBr_3_ can be performed at room temperature, which may be due to the higher Lewis acidity.

**Scheme 31:**
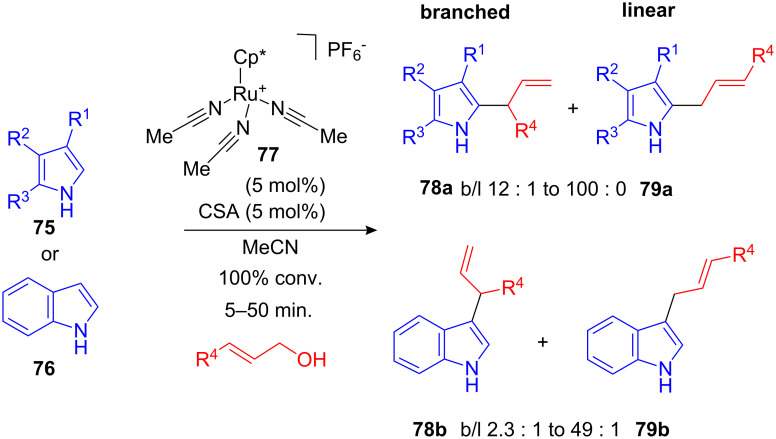
Ru(IV)-catalyzed allylation of indole and pyrroles with unique regioselectivity.

While the catalysts described above gave primarily linear allylation products, procedures that would give branched allylated arenes would be even more desirable. An example of such a transformation was recently uncovered by Pregosin and co-workers. The cationic Trost-type Ru(IV)-sulfonate catalyst **77** gives high regioselectivity for branched allylated arenes. As electron-rich arenes, pyrroles **75** and indole (**76**) can be utilized (Scheme 31) [86]. Interestingly, the addition of sulfonic acid was crucial for the high reactivity and the observed regioselectivity [87–88]. Branched/linear ratios of the desired products **78** and **79** were found to range between 2.3 : 1 and 100 : 0. The selectivity of this reaction is thought to be driven by the LUMO of the Ru(IV)-allyl complex intermediate.

**Scheme 32:**
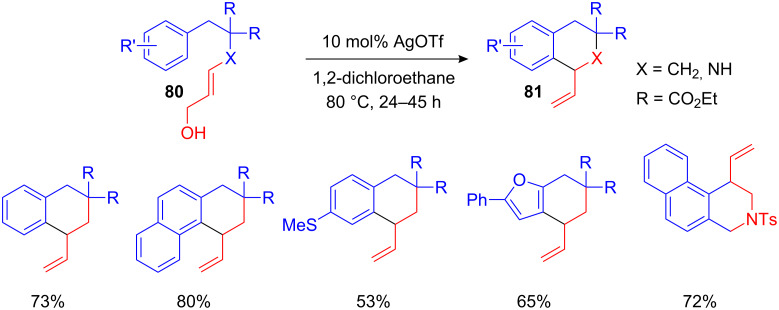
Silver(I)-catalyzed intramolecular FC-type allylation of arenes and heteroarenes.

Besides intermolecular allylations, intramolecular FC-type allylations are of great importance. One of the first intramolecular FC-type transformations using allyl alcohols was developed by Nishizawa et al. using Hg(OTf)_2_ as Lewis acid [89]. Since Hg(II) is not an environmentally friendly transition metal, Bandini et al. developed a “greener” Ag(I)- and Mo(II)-catalyzed variant (Scheme 32) [90–91]. The desired tetrahydronaphthalenes **81** were obtained in high yields under ambient reaction conditions (Scheme 32) [91]. Subsequently a very similar iron(III)-catalyzed intramolecular FC alkylation of propargyl alcohols was developed by Zhou et al. Depending on the reaction conditions one observes either the corresponding dihydronaphthalenes or allenes as the main reaction products [92].

### Catalytic propargylation of arenes

**Scheme 33:**
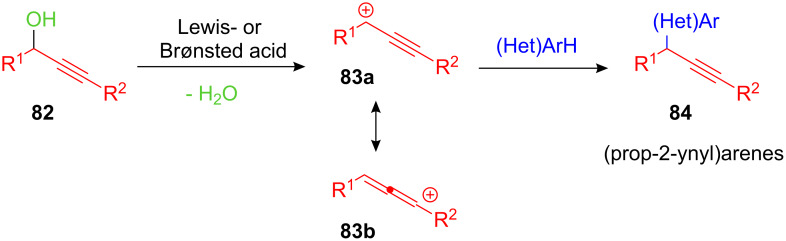
FC-type alkylations of arenes using propargyl alcohols.

(Prop-2-ynyl)arenes **84** are widely distributed structural motifs in organic chemistry due to the high synthetic value of the alkyne functionality. This makes them suitable precursors for the synthesis of highly substituted 1,1-diarylalkanes. Thus efficient routes to this important core structure are constantly needed. One of the most efficient ways for synthesising (prop-2-ynyl)arenes is the direct propargylation of arenes and heteroarenes with propargyl alcohols. This reaction is typically more difficult to achieve since the intermediary propargyl cations **83a** are in equilibrium with the corresponding sp^2^-hybridized allenylium cations **83b** and the latter are prone to undergo polymerization or side reactions, such as γ-substitution (Scheme 33).

So far the Nicholas reaction has been the method of choice for the propargylation of aromatic systems. However the use of stoichiometric amounts of Co_2_(CO)_8_ and oxidation reagents denotes a fairly inefficient transformation [93].

**Scheme 34:**
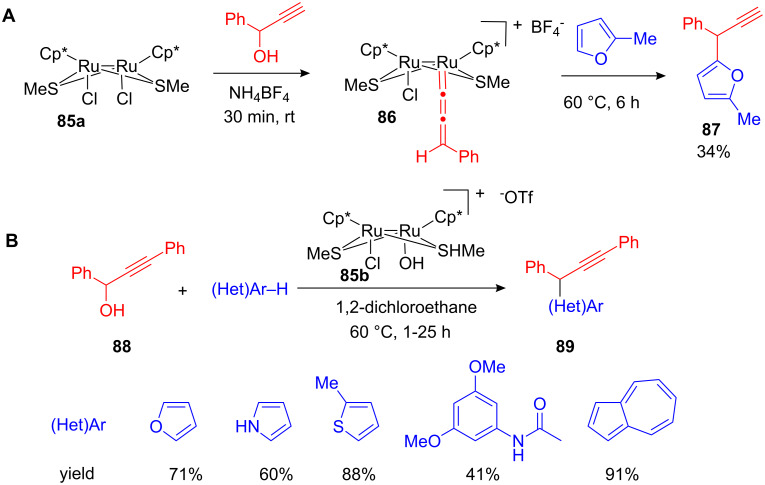
**(A)** Propargylation of arenes with stoichiometric amounts of the Ru-allenylidene complex **86**. **(B)** First catalytic propargylation of (hetero)arenes using a cationic Ru-complex.

Uemura and co-workers discovered in 2002 that heating stoichiometric amounts of a cationic Ru-allenylidene complex **86**, which has been synthesised from the Ru-precursor **85a**, together with 10 equiv of 2-methylfuran led to rapid formation of 5-propargylated 2-methylfuran **87** in 34% yield (Scheme 34A) [94]. Subsequently a catalytic variant of this transformation was developed by using diruthenium complexes **85a** and **85b** (Scheme 34B) [95]. The reaction proceeded smoothly and yields of the propargylated arenes **89** were even higher when only small amounts of the catalyst were used.

**Scheme 35:**
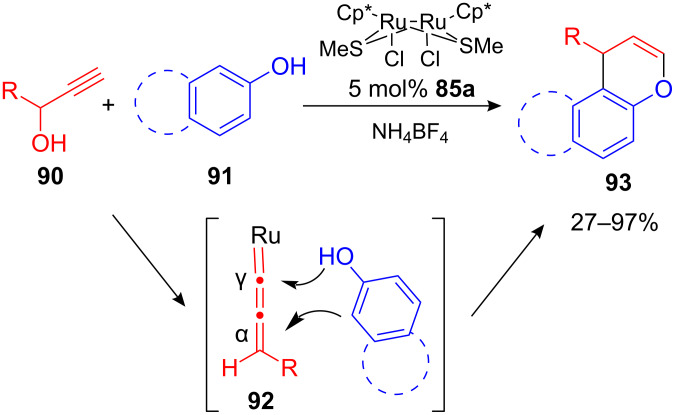
Diruthenium-catalyzed formation of chromenes and 1*H*-naphtho[2,1-*b*]pyrans.

Interestingly, if phenols and naphthols **91** were applied as nucleophiles, both C_α_- and C_γ_-carbon atoms of allenylidene-Ru **92** complex were attacked giving rise to the corresponding chromenes and 1-substituted 1*H*-naphtho[2,1-*b*]pyrans **93**, respectively, in moderate to excellent yields (Scheme 35). Electron-withdrawing as well as electron-donating functional groups were tolerated on the phenol scaffold, whereas 1,1-diaryl-substituted propargylic alcohols did not react at all [96]. The same authors expanded their procedure to an enantioselective propargylation of arenes using a chiral Ru-complex [97]. This outstanding transformation is discussed in more detail in the following chapter of this review.

A related cationic Ru-vinylidene complex was used for an efficient alkynylation of pyridines with (alkyn-1-yl)trimethylsilane [98].

Based on these preliminary results, various catalytic propargylations of arenes have been developed, employing catalysts such as Mo/chloranil [16], polymolybdophosphoric acid supported silica [99], TsOH [100], FeCl_3_ [101–102], BiCl_3_ [103], Sc(OTf)_3_ [104] other Ru- or [105–107], Re-complexes [108] and even molecular iodine [109].

**Scheme 36:**
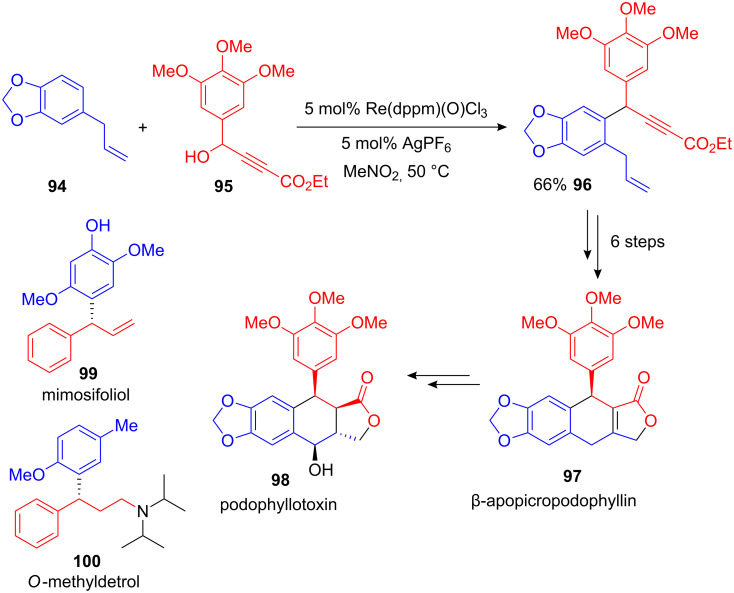
Rhenium(V)-catalyzed FC propargylations as a first step in the total synthesis of podophyllotoxin, mimosifoliol and *O*-methyldetrol.

Toste and co-workers developed a mild rhenium-catalyzed propargylation of electron-rich arenes. In addition to principal method development, the authors applied their procedure towards the synthesis of several biologically active molecules. Starting from ethyl propiolate **95** and safrole **94**, the desired FC alkylation product **96** was isolated in 66% yield. A subsequent six-step synthesis yielded β-apopicropodophyllin (**97**), a previously described precursor of cyctotoxic aryltetralinlactone podophyllotoxin (**98**; Scheme 36) [108].

Likewise, the two natural occurring benzhydryl compounds mimosifoliol **99** and *O*-methyldetrol **100** were obtained from readily available arenes and propargyl alcohols. The FC propargylations that were necessary in the first step were carried out in the presence of air and moisture with just 5 mol% Re(O)(dppm)Cl_3_ and 5 mol% potassium or silver hexafluoridophosphate as a halide abstractor. Remarkably, γ-heteroatom substituted propargyl alcohols can also be used as alkylating reagent.

**Scheme 37:**
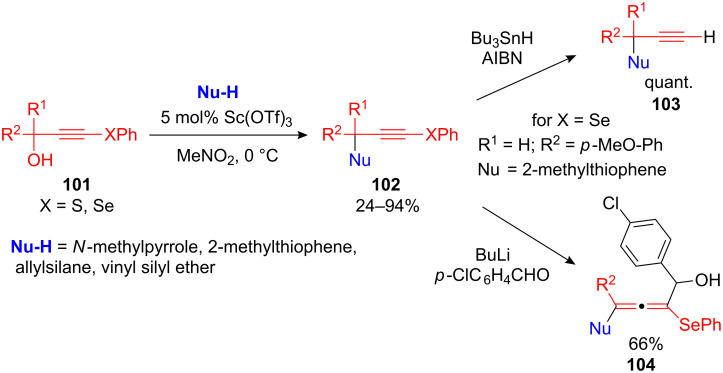
Scandium-catalyzed arylation of 3-sulfanyl- and 3-selanylpropargyl alcohols.

Yoshimatsu et al. developed a scandium-catalyzed FC alkylation using 3-sulfanyl- and 3-selanylpropargylic alcohols **101**. With 5 mol% of Sc(OTf)_3_ as the catalyst and a surprisingly low reaction temperature (0 °C) the desired propargylated arenes **102** were isolated in good to high yields. Beside arenes and heteroarenes, allylsilane and vinyl silyl ethers were used as nucleophiles in this transformation [110]. The substituted γ-selenopropargyl compounds were readily transformed into the terminal alkynes **103** by treatment with tributyltin hydride or were further functionalized with aldehydes to form the highly substituted allenyl alcohols **104** (Scheme 37).

**Scheme 38:**
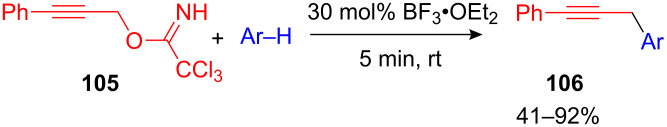
Synthesis of 1,3-diarylpropynes via direct coupling of propargyl trichloracetimidates and arenes.

While the methods described above used α-arylated propargyl alcohols as highly reactive alkylating reagents, reports of the arylation of α-unsubstituted propargyl groups are few. In these reactions the alcohol has to be activated as a leaving group in order to obtain sufficient reactivity. One example utilizing the propargyl trichloracetimidates **105** was described recently by Wang and co-workers. With 30 mol% of boron trifluoride etherate, the highly desired 1,3-diarylpropynes **106** were obtained in good yields. The reaction was over within 5 min and various arenes and heteroarenes, including toluene, xylene, thiophene or furan were tolerated as nucleophiles (Scheme 38) [111].

### Catalytic enantioselective and diastereoselective Friedel–Crafts alkylations

**Scheme 39:**
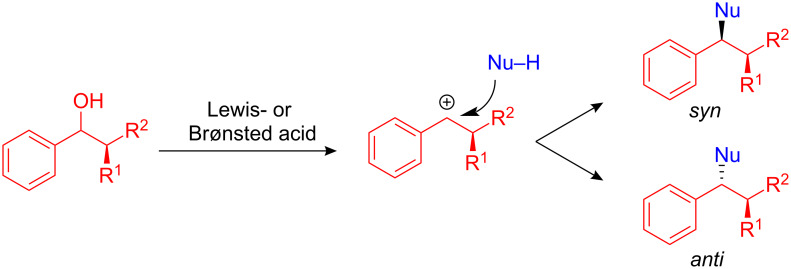
Diastereoselective substitutions of benzyl alcohols.

Despite the recent effort in the development of FC alkylations, enantio- and diastereoselective FC alkylations using chiral alcohol precursors are rare. Due to the fact that Lewis- or Brønsted-acid-catalyzed FC alkylations are passing through a carbocationic intermediate, the alcohol itself can not be used as the chiral precursor (Scheme 39). In order to circumvent this problem, benzyl alcohols with a stereocenter in the α-position were employed as directing groups.

**Scheme 40:**
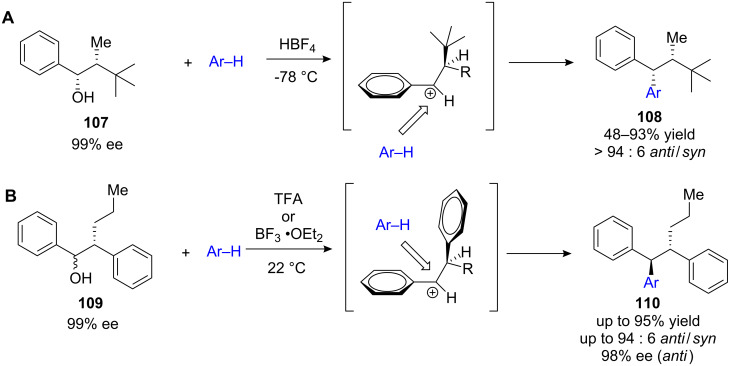
**(A)** First diastereoselective FC alkylations developed by Bach et al. **(B)** *anti*-Selective FC alkylation of α-phenyl substituted benzyl alcohols.

This approach was used by Bach and co-workers employing the α-*tert*-butyl-substituted benzyl alcohol **107** (Scheme 40A). Various electron-rich arenes and heteroarenes were applied giving rise to chiral arenes **108** with remarkable *syn*-diastereoselectivities of up to 94 : 6 d.r. [112–113]. Beside the *tert*-butyl moiety as regio-discriminating group, other useful synthetic functional groups such as nitro-, cyano- or hydroxy functionalities could effectively be used [114]. Enantiomerically enriched benzyl alcohols did not lose their stereochemical information during substitution. In addition both, *syn*- and *anti* diastereoisomer of the starting material gave the *syn*-configured products as the major diastereoisomer. This strongly indicates a carbocation as reaction intermediate and rules out an S_N_1-type reaction mechanism. Additionally, low temperature NMR studies in superacidic media clearly profed the carbocationic character of the benzylic carbon atom [113].

When the *tert*-butyl-group is replaced by phenyl functionalities **109**, a reverse in diastereoselectivity is observed. This may be a result of п-stacking interactions [115]. Again, various arenes and heteroarenes, including indoles, thiophenes, pyrroles or furans gave the desired *anti*-1,1,2-triarylalkanes **110** in good yields and with high diastereoselectivities (Scheme 40B). BF_3_•OEt_2_ and TFA were used in stoichiometric amounts to promote this reaction. However, application of BF_3_ resulted in significantly higher reactivity, while the diastereoselectivity was higher when TFA was used. The enantiomeric excess did not significantly diminish during this procedure, which makes this reaction a convenient and efficient route to optically pure 1,1,2-triarylalkanes.

**Scheme 41:**
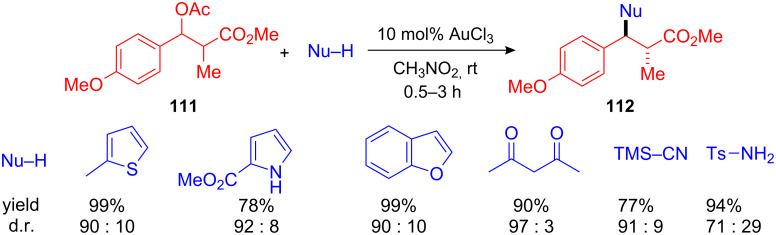
Diastereoselective AuCl_3_-catalyzed FC alkylation.

A drawback of this first diastereoselective FC alkylation of chiral benzyl alcohols was the necessity for stoichiometric amounts HBF_4_ and low temperatures in order to observe high diastereoselectivities. To overcome these drawbacks, Bach et al. developed an efficient AuCl_3_-catalyzed arylation of benzyl alcohols and acetates with chiral phenylbutanoates **111** [116]. In addition to arenes and heteroarenes, other nucleophiles including allylsilane, tosylamine, TMS-cyanide, acetylacetonates and silyl enol ethers were successfully used as nucleophilic components giving the substituted 3-phenylbutanoates **112** in high yields and with excellent diastereoselectivities (Scheme 41). The reaction could be performed at ambient temperatures and in sharp contrast to the previous described HBF_4_-mediated procedure, high *anti*-selectivity was observed in the formation of **112**.

**Scheme 42:**
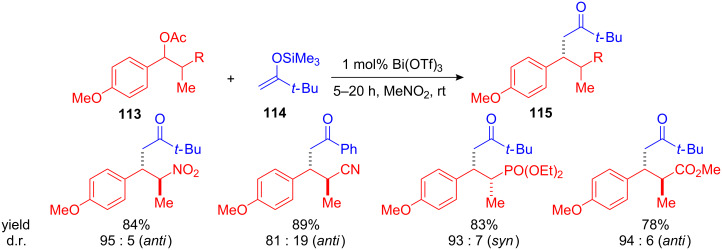
Bi(OTf)_3_-catalyzed alkylation of α-chiral benzyl acetates with silyl enol ethers.

Similar methods using silyl enol ethers were subsequently developed involving Bi(OTf)_3_ as Lewis acid catalyst [117–118]. While bismuth-catalyzed arylation of benzyl alcohols proceeded only at temperatures between 55 and 100 °C, the reaction of *p*-methoxybenzyl acetates **113** with silyl enol ethers **114** took place even at ambient temperatures to give the desired products **115** (Scheme 42) [118]. Again different chiral benzyl acetates were efficiently substituted employing low amounts of catalyst (1–5 mol%). While nitro-, cyano- and methyl ester derivatives gave remarkable *anti*-selectivity, the corresponding α-phosphonate showed high *syn*-selectivity. In general, the diastereoselectivity can be explained by comparing the A-values (an estimation of the steric demand) of the α-functional groups. If the A-value of the functional group is higher than the A-value of the methyl group, *Re*-attack is favoured giving the corresponding *anti* products and *vice versa* [119].

**Scheme 43:**
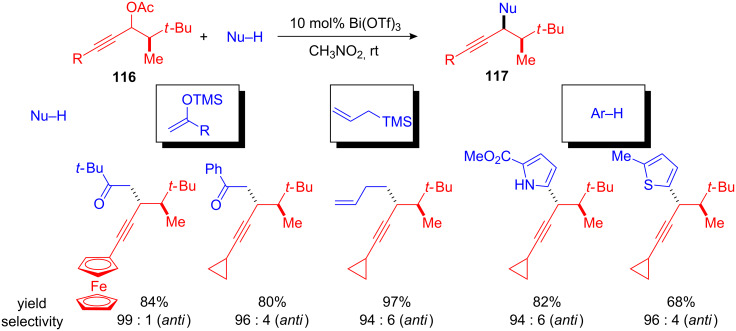
Bi(OTf)_3_-catalyzed diastereoselective substitution of propargyl acetates.

In addition to benzyl alcohols and propargyl alcohols, acetates can also be used in diastereoselective FC alkylations. Again Bi(OTf)_3_ was the Lewis acid of choice. With 10 mol% of catalyst the α-*tert*-butyl-substituted propargyl acetates **116** could be substituted with silyl enol ethers, allylsilane and various arenes and heteroarenes to give the desired alkylated alkynes **117** in high yields and excellent *anti* diastereoselectivities of up to 99 : 1 d.r. (Scheme 43) [117].

Treatment of chiral β-hydroxy esters with aromatic and aliphatic nitriles in the presence of catalytic amounts of TfOH and subsequent hydrolysis in a Ritter-type reaction led to the desired *anti* α-amino esters with high diastereoselectivities [120].

**Scheme 44:**
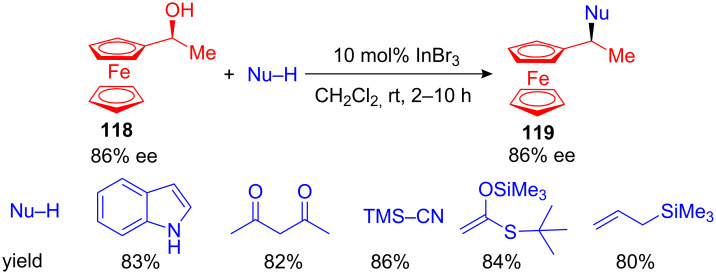
Nucelophilic substitution of enantioenriched ferrocenyl alcohols.

Next to the described developments, Cozzi et al. reported that enantioenriched ferrocenyl alcohols **118** can be efficiently substituted with various C-nucleophiles. Applying 10 mol% of InBr_3_ the desired substituted ferrocenyl derivatives **119** were isolated in high yields under ambient conditions (Scheme 44). The configuration was retained during the nucleophilic substitution. The optically active ferrocenyl alcohols can be easily synthesised from the corresponding ferrocenyl ketone precursor via an enantioselective aminoindanol/BH_3_ reduction [121]. The same authors reported shortly after their initial finding a highly efficient substitution of ferrocenyl alcohols in water, without the need of any Lewis-acid catalyst [50]. A comparable, non-chiral version of this C–C bond forming reaction using ceric ammonium nitrate has recently been developed [122].

**Scheme 45:**
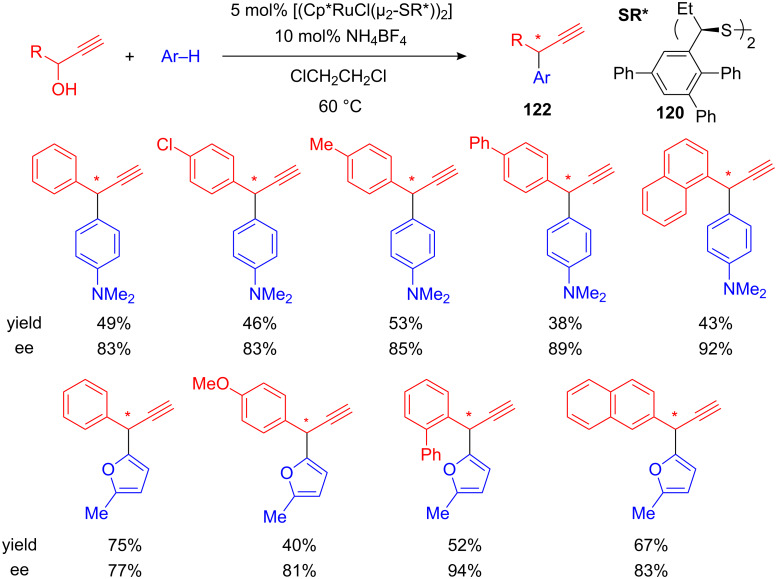
First catalytic enantioselective propargylation of arenes.

A first catalytic enantioselective Friedel–Crafts alkylation with propargyl alcohols as electrophile has been developed by Nishibayashi and co-workers. Chirality was introduced by a thiolate-bridged diruthenium complex which is based on optically active disulfide ligand **120**. 2-Alkylfurans as well as *N,N*-dimethylaniline were efficiently utilized as nucleophiles and the corresponding highly valuable optically active propargylated aromatic compounds **122** were isolated in moderate yield but with excellent enantioselectivities of up to 94% ee (Scheme 45) [97].

This milestone in the catalysis of the Friedel–Crafts alkylation should be considered a good starting point for the development of other catalytic enantioselective FC alkylations, using not only propargyl- but also allyl- or benzyl alcohols as electrophilic components.

## Future Perspectives

Despite the great efforts that have been undertaken in the last decade with catalytic FC alkylations, there are still major challenges that need addressing. To the best of our knowledge no catalytic enantioselective Friedel–Crafts reactions leading to enantioenriched 1,1-diarylalkanes are known. Chiral, bifunctional Lewis- or Brønsted acid catalysts are required that stabilize the transient carbocations and are able to mediate enantioselective S_N_1 reactions.

Moreover, Friedel–Crafts alkylations of arenes bearing free amines or nitrogen-containing heterocycles will be a great advance in this area. This increase in functional group tolerance would allow the use of the Friedel–Crafts reactions in the late stages of complex natural product synthesis or in the preparation of biological relevant molecules, including pharmaceuticals and agrochemicals. Furthermore, the extension of substrate scope away from π-activated alcohols and double bonds to less reactive alkyl alcohols is a major goal, and will finally eliminate the need for haloalkanes in FC alkylations.
